# The effect of simulation-based training on initial performance of ultrasound-guided axillary brachial plexus blockade in a clinical setting – a pilot study

**DOI:** 10.1186/1471-2253-14-110

**Published:** 2014-11-26

**Authors:** Owen O’Sullivan, Gabriella Iohom, Brian D O’Donnell, George D Shorten

**Affiliations:** Department of Anaesthesia and Intensive Care Medicine, Cork University Hospital, Wilton, Cork Ireland; Department of Anaesthesia and Intensive Care Medicine, University College Cork, Cork, Ireland; ASSERT for Health Centre, University College Cork, Cork, Ireland

**Keywords:** Ultrasound-guided regional anesthesia, Simulation, Validation, Virtual reality, Procedural training, Technology-enhanced learning

## Abstract

**Background:**

In preparing novice anesthesiologists to perform their first ultrasound-guided axillary brachial plexus blockade, we hypothesized that virtual reality simulation-based training offers an additional learning benefit over standard training. We carried out pilot testing of this hypothesis using a prospective, single blind, randomized controlled trial.

**Methods:**

We planned to recruit 20 anesthesiologists who had no experience of performing ultrasound-guided regional anesthesia. Initial standardized training, reflecting current best available practice was provided to all participating trainees. Trainees were randomized into one of two groups; (i) to undertake additional simulation-based training or (ii) no further training. On completion of their assigned training, trainees attempted their first ultrasound-guided axillary brachial plexus blockade. Two experts, blinded to the trainees’ group allocation, assessed the performance of trainees using validated tools.

**Results:**

This study was discontinued following a planned interim analysis, having recruited 10 trainees. This occurred because it became clear that the functionality of the available simulator was insufficient to meet our training requirements. There were no statistically significant difference in clinical performance, as assessed using the sum of a Global Rating Score and a checklist score, between simulation-based training [mean 32.9 (standard deviation 11.1)] and control trainees [31.5 (4.2)] (p = 0.885).

**Conclusions:**

We have described a methodology for assessing the effectiveness of a simulator, during its development, by means of a randomized controlled trial. We believe that the learning acquired will be useful if performing future trials on learning efficacy associated with simulation based training in procedural skills.

**Trial registration:**

ClinicalTrials.gov identifier: NCT01965314. Registered October 17th 2013.

**Electronic supplementary material:**

The online version of this article (doi:10.1186/1471-2253-14-110) contains supplementary material, which is available to authorized users.

## Background

The learning environment in which resident anesthesiologists acquire procedural skills has fundamentally changed. Training programmes are shorter and afford fewer training opportunities. Patient, institutional and regulatory expectations limit acceptance of trainees acquiring skills by "practicing" on patients. In the context of ultrasound-guided axillary brachial plexus blockade (USgABPB), we have demonstrated that anesthesiologists in Ireland perceive a lack of learning opportunity as being the most important impediment to procedural skill development [1].

It is indisputable that simulation will play in an increasingly important part in the training and assessment of procedural skills [2]. Simulation offers trainees an opportunity to hone skills in a risk-free environment. Training bodies are attempting to move from traditional time-based training programmes to competency-based training [3]. Since January 2010, the American Board of Anesthesiology (ABA) has included simulation-based training as a mandatory component of Maintenance of Certification in Anesthesiology (MOCA) [4]. A recent meta-analysis demonstrated that technology-enhanced simulation-based training is associated with large positive effects on knowledge, skills, and behaviors, and moderate effects on patient based outcomes [5].

To date, simulation in ultrasound-guided regional anesthesia (UGRA) has largely been limited to tissue (e.g. turkey breasts or cadavers) and non-tissue (e.g. gelatin or tofu) phantoms [6, 7]. Computer-based VR simulation has been utilized effectively for training in a number of procedural domains, e.g. laparoscopic surgery [8] and colonoscopy [9]. Grottke et al. [10] have previously described the development of a virtual reality (VR) simulator for regional anesthesia guided by peripheral nerve stimulation. Previous work at our institution described the development of a similar device simulating spinal anesthesia [11]. VR simulation offers a number of advantages over the alternatives; (i) variety of predefined standardized scenarios, (ii) multiple anatomical variations, (iii) models do not degrade with repeated needle insertion, (iv) realistic representations of anatomy acquired via MRI, CT or ultrasound derived data, (v) normal variation of a single anatomical site can be represented, and (vi) multiple anatomical sites (thus different types of blocks) can be represented in a single simulator [12]. We have participated in developing a VR visuo-haptic simulator to train USgABPB, as part of a collaborative project with the National Digital Research Centre ( http://www.ndrc.ie). The simulator is intended to render the haptic (related to tactile and proprioceptive) sensations normally felt during manipulation of both needle and ultrasound probe. We set out to assess the effect of training USgABPB utilizing a novel prototype simulator on skill transfer, during its development.

We hypothesized that VR-based training offers an additional learning benefit over standard training (using cadaveric dissection and human volunteers) in preparing novice anesthesiologists to perform their first USgABPB in the clinical setting. We carried out pilot testing of this hypothesis using a prospective, single blind, randomized control trial.

## Methods

This prospective, randomized controlled trial was conducted at Cork University Hospital and St Mary’s Orthopaedic Hospital (Cork, Ireland). The Clinical Research Ethics Committee of the Cork Teaching Hospitals approved the study and the study was registered with ClinicalTrials.gov (NCT01965314). All subjects, patients and anesthesiologists, provided written informed consent. We planned to recruit 20 residents (all College of Anaesthetists of Ireland affiliated trainees) with no experience of performing UGRA. The sample size was arbitrarily based on previous studies indicating the effectiveness of VR simulation-based teaching procedural skills to novices [8]. Subjects provided baseline personal data, experience in practice of anesthesia (years in training) and handedness. Each subject was asked to categorize his/her (i) previous experience of peripheral nerve blockade with peripheral nerve stimulation [0 = 0 blocks, 1 = 1–5 blocks, 2 = 5–10 blocks, 3 = 10–50 blocks, 4 = 50–100 blocks, 5 ≥ 100 blocks] (ii) previous experience of ultrasound-guided vascular access [0 = 0 procedures, 1 = 1–5 procedures, 2 = 5–10 procedures, 3 = 10–50 procedures, 4 = 50–100 procedures, 5 ≥ 100 procedures] (iii) previous attendance at a peripheral nerve blockade course (incorporating ultrasound-guided techniques) [0 = never, 1 = ≤half day course, 2 = full day course, 3 = ≥2 day course, 4 = multiple courses]. Baseline visuo-spatial ability was assessed using the card rotation, shape memory, and snowy picture tests (Educational Testing Service) [13]. Psychomotor ability was assessed using a grooved pegboard (Lafayette Instruments, Lafayette, IN). Subjects were randomly allocated (non-stratified) into 1 of 2 groups, (i) the control group (CG) or (ii) the simulator trained group (SG) using random number tables.

### Common training

All participating anesthesiologists received standardized training. These educational sessions took place in the Department of Anatomy, University College Cork. The educational sessions were attended by 4–6 trainees. A single anesthesiologist (BOD) with expertise in both teaching and performing the procedure delivered all sessions and supervised the trainees during the hands-on sessions. Each session comprised a number of components, namely; (i) a didactic session, (ii) an hands on session with appropriately prepared cadaveric specimens, (iii) ultrasound scanning of a volunteer, and (iv) a needling skills session with tissue phantoms. Subjects were taught to perform USgABPB using a technique as described in Appendix IV and V of ‘The American Society of Regional Anesthesia and Pain Medicine and the European Society of Regional Anaesthesia and Pain Therapy Joint Committee Recommendations for Education and Training in Ultrasound-Guided Regional Anesthesia’ [14]. All ultrasound examinations performed on volunteers or on patients entailed the use of a Sonosite M Turbo (Sonosite, Bothell, WA, USA) (or similar device) with a 7–12 MHz 38 mm linear probe. Following the educational intervention, all subjects were asked to give written feedback, by means of a standard form, on the content and delivery of the session. On completion of the common training those in the CG received no further training and those in the SG went on to complete a proficiency-based training period using a prototype simulator.

### Simulator training

The simulator was comprised of two PHANTOM Desktop devices ( http://www.sensable.com, Wilmington, MA, USA), a desktop computer (Hewlett-Packard, http://www.hp.com), a liquid crystal display (LCD) monitor (Samsung Sync master 2233) capable of rendering 120 frames per second synchronized with a pair of 3D stereoscopic glasses ( http://www.nvidia.co.uk), and the H3D API ( http://www.sensegraphics.se). The SG subjects were asked to scan and perform procedure specific tasks on a virtual arm. The model of the arm was informed using 1.5 Tesla MRI DICOM datasets which generated skin and bone surfaces. A number of computer generated structures were added to this model based on typical anatomical positioning (The Science Picture Company, http://www.sciencepicturecompany.com, Dublin, Ireland). These were the axillary artery and three nerves (representing median, ulnar and radial nerves). The resultant image was thus a computer generated "animation".

Before subjects began simulation-based training, 3 experts (each of whom had undertaken structured higher subspecialty training in regional anesthesia and maintained proficiency by performing at least 100 UGRA procedures during the previous year) performed each task under similar conditions on three consecutive occasions. The mean values of their performances set a proficiency level against which subsequent trainee performance was benchmarked.

**Figure 1:**
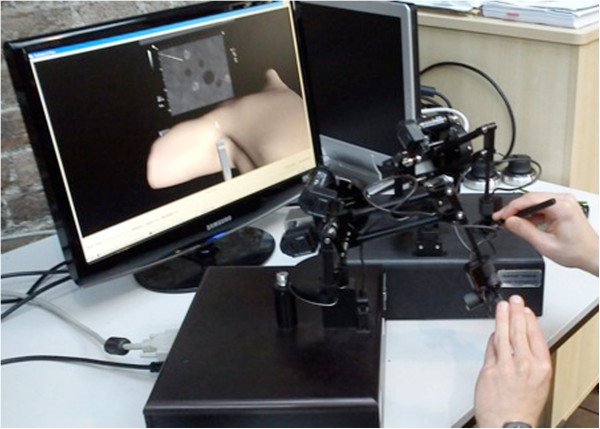
**Configuration of simulator similar to that during trial.**

**Figure 2:**
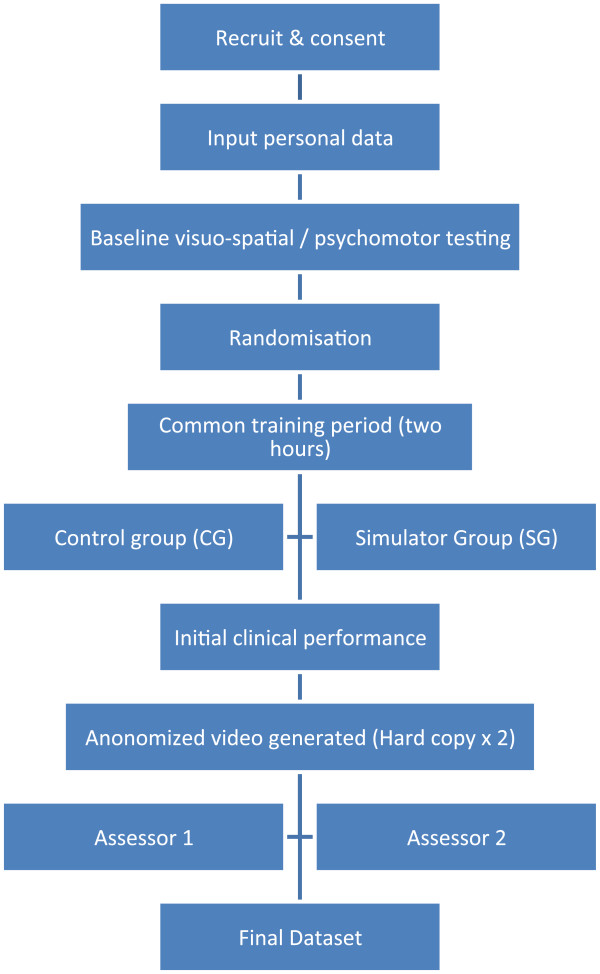
**Study flow diagram.**

**Figure 3:**
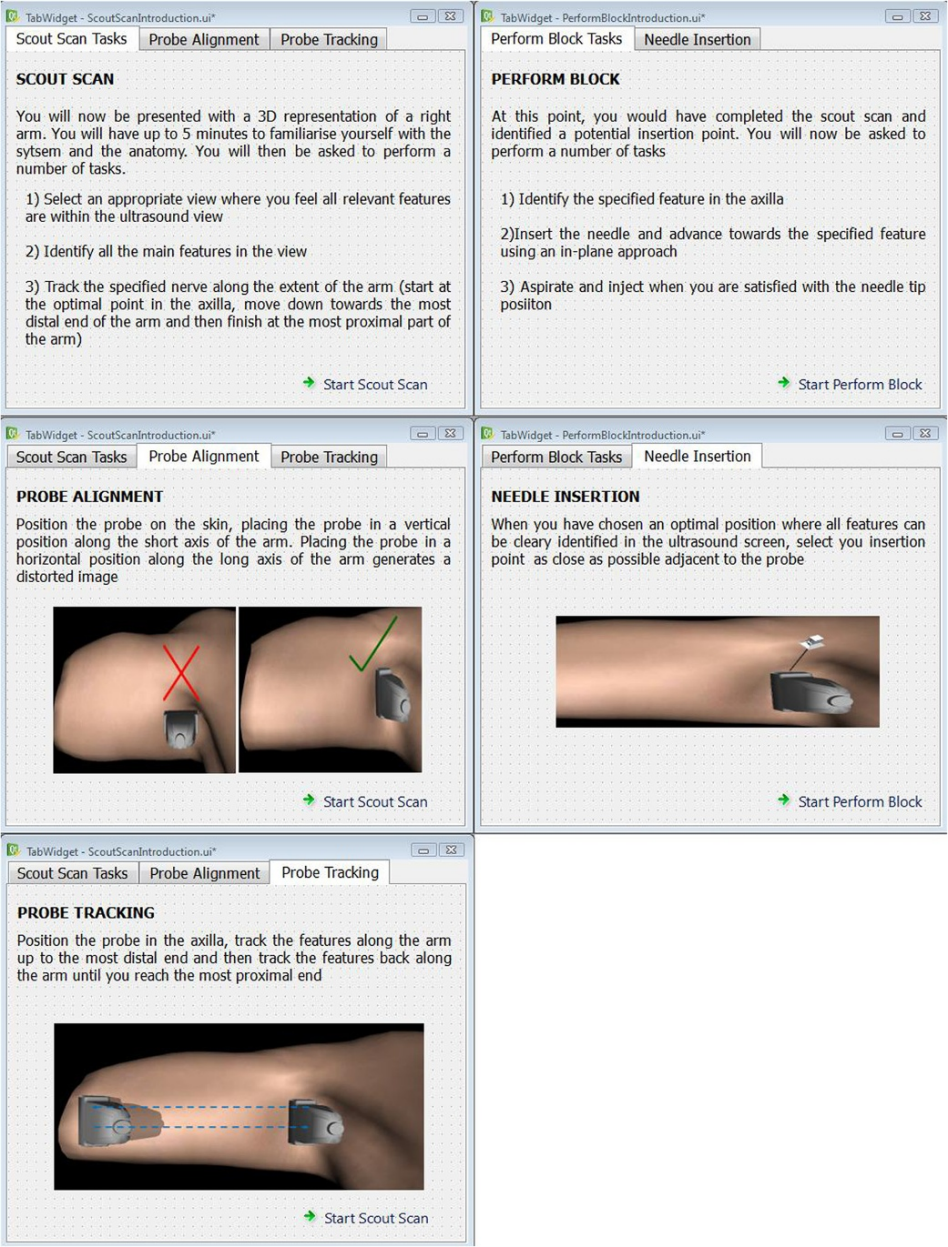
**Sample instruction material.**

**Figure 4:**
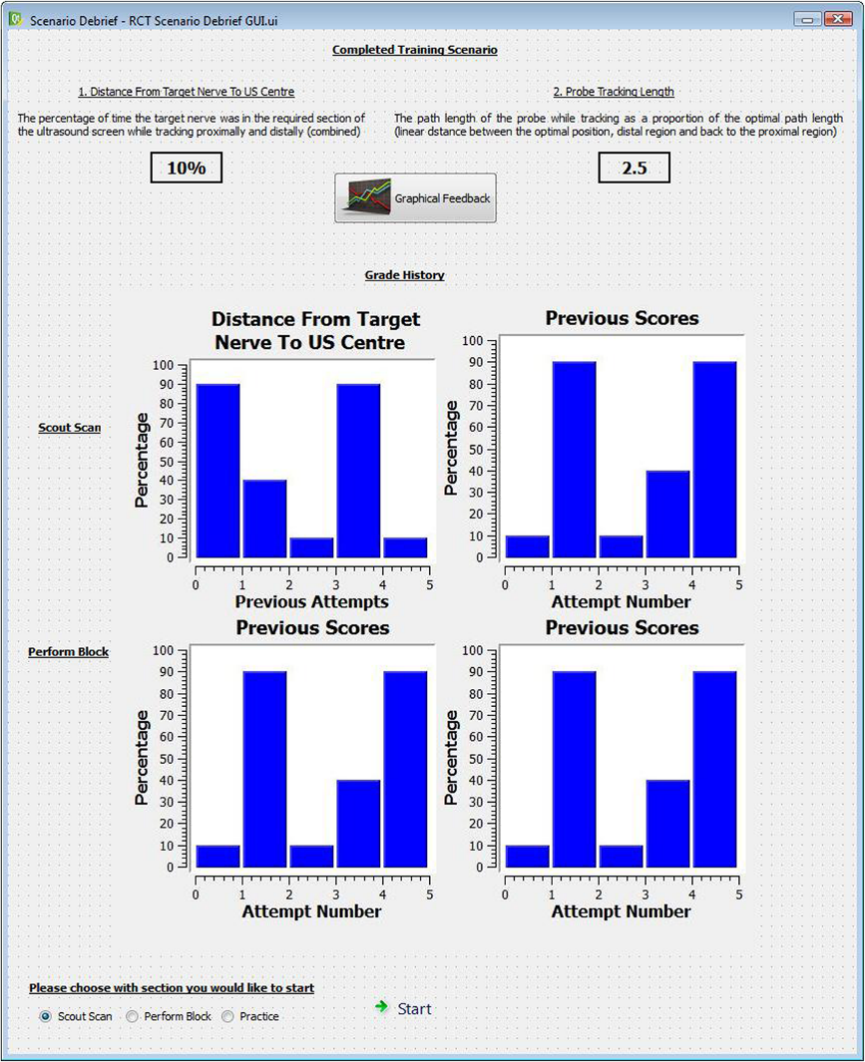
**Sample end of session debrief.**

**Figure 5:**
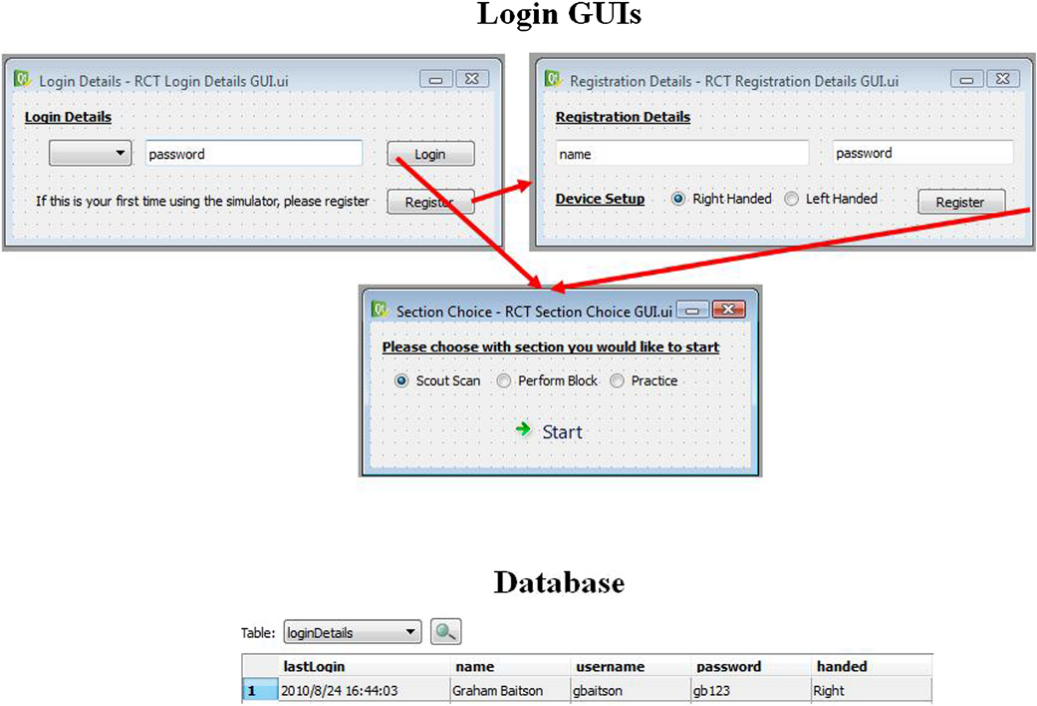
**Automated login process.**

Following initial familiarization with the simulator, lasting 50 – 60 minutes (duration partially due to the prototypal nature of the device), SG subjects were asked to complete 4 procedure specific tasks to a predefined proficiency level, 2 relating to ultrasound scanning (utilizing a single haptic device) and 2 relating to needle advancement under ultrasound guidance (concurrently controlling two haptic devices – see Figure 1). Computer generated feedback was given to the subject after each attempted performance of each task. Participants were required to meet proficiency levels on two consecutive attempts before passing each task. In order to complete simulation training the SG participants had to pass all 4 tasks. The tasks were specifically chosen to cover the pre-procedural scout scan and the needling component of USgABPB, while also permitting capture of behaviors likely to lead to significant clinical errors [15]. Table 1 outlines each task, the feedback given and the proficiency level which had to be met. There was no specified time limit to meet these requirements. Subjects were free to control the frequency and duration of use of the simulator. Following initial orientation, training on the simulator in this study was largely unsupervised. An investigator was immediately available to address any technical issues which may have arisen. A flow diagram of the study design is provided (Figure 2). Figures 3, 4, and 5 provide sample images, representative of (i) instructional material, (ii) automated feedback, and (iii) the automated login process.

**Table 1:** **Task, the feedback given and the proficiency level to be met**

	Task	Feedback	Proficiency Level
**1**	Identify the 4 relevant structures represented at a point in the axilla	Number of structures correctly identified	All four structures identified
**2**	Follow the course of two of these structures (median and ulnar nerves) from axilla towards the elbow, while keeping the structures in the centre of the virtual ultrasound screen	The amount (%) of the structure represented in the middle of the virtual ultrasound as a proportion of the total length of the structure (from axilla to elbow) (out of 100%)	Mean expert performance
**3**	Advance a virtual needle towards a specified target (median nerve) keeping the needle in plane during advancement	The proportion (%) of needle advancement which occurred "in plane" as a proportion of the total distance the needle tip advanced in the virtual arm	Mean expert performance
**4**	Trigger a virtual injectate at an appropriate distance from the target.	The distance from the needle tip to the target structure when injection triggered	Injection at a distance not less than the mean expert minimum distance and not more than the mean expert maximum distance. Needle tip must also be visualized at the time of triggering.

### Assessment

We aimed to assess the performance of the subjects first USgABPB within two weeks of completing the educational interventions. Patients recruited required anesthesia for forearm/wrist/hand surgery where USgABPB would ordinarily be offered as standard care. Informed patient consent was obtained. Study participant performance of USgABPB was closely supervised by a trained regional anesthesiologist. Intravenous sedation was administered as clinically indicated, and subsequent care of the patient may have included general anesthesia. Subjects were asked to perform USgABPB using an in-plane approach and short-axis view. The procedure was video recorded using a handheld video recording device (Flip Ultra, http://www.theflip.com) in a manner aimed to conceal both the identity of the patient and the identity of the anesthesiologists performing the block. Two experts in UGRA later evaluated the video data.

### Patient inclusion and exclusion criteria were

Inclusion criteria: ASA grades I and II, age 18–80 years, capacity to consent, already consented for USgABPB, Body Mass index 20 – 26 kg/m^2^

Exclusion criteria: Parameters outside inclusion criteria, contraindication to regional anesthesia, language barrier, psychiatric history, pregnancy.

### Outcome measures

The subject’s performances were assessed retrospectively based on a task specific, dichotomous, checklist and a behaviorally anchored 5-point global rating scale previously validated for this procedure (see Additional file 1) [16]. Two experts, experienced with this form of evaluation, carried out these assessments. The experts were blinded to group allocation. The primary outcome measure was the average value of the sum of (i) global rating scale (GRS) scores and (ii) total number of procedural checklist items as assessed by the two blinded experts. Secondary outcome measures were (i) GRS scores, (ii) checklist scores, (iii) procedural times (iv) number of needle passes, (v) block success (as defined by sensory and motor blockade in the distribution of all four relevant nerves demonstrated within 15 minutes of USgABPB), (vi) block failure (as defined by an unanticipated need for an additional peripheral nerve block or an unplanned conversion to general anesthesia), (vii) participating anesthesiologist confidence levels (measured on a ten point verbal rating scale, on completion of assessment of the block – "How confident were you in performing the block?") following performance of the USgABPB, and (viii) patient satisfaction measure (measured on a ten point verbal rating scale, on discharge from recovery "How satisfied were you with the block?").

SPSS version 17.0.2 software (SPSS, Inc., Chicago, IL, USA) was used for data analysis. Data were analyzed using Mann–Whitney’s U-test for continuous variables. A p value of <0.05 was considered significant. Inter-rater levels of agreement were estimated using Cohen’s Kappa and percentage inter-rater reliability, defined as agreements/(agreements + disagreements) times 100 [8].

## Results

Having originally planned to recruit 20 trainees, this study was discontinued following a planned interim analysis. Ten trainee anesthesiologists were recruited from a university affiliated teaching hospital (Cork University Hospital) in July 2010, 4 to the Simulation group and 6 to the Control group Recruitment was discontinued because, it became clear that the functionality of the available simulator was insufficient to meet our training requirements. Baseline participant data are summarized in Table 2. The results of visuo-spatial testing using Snowy Picture, Shape Memory and Card Rotation Tests and psychomotor assessment using the Perdue Pegboard are summarized in Table 3. Trainees in the SG did score significantly better in the Shape Memory Test than those in the CG, a measure of visual memory (23.3 (4.6) vs. 12.3 (4.6), p = 0.010). The differences in other visuo-spatial and psychomotor tests were not statistically significant.

**Table 2:** **Baseline participant data**

	Simulation group (n = 4)	Control group (n = 6)
Male: Female	2: 2	5: 1
Years Experience in practice of anesthesia [*Median(Range)*]	5(0–12)	4.5(0–22)
Previous Experience of Peripheral Nerve blockade with peripheral nerve stimulation	0.5(0–4)	1.5(0–3)
Previous Experience of Ultrasound-Guided Vascular Access	2(0–4)	1(0–5)
Previous Attendance at a Peripheral Nerve Blockade course (incorporating Ultrasound-Guided techniques)	0.5(0–2)	0(0–3)
Handedness	3 Right +1 Ambidextrous	6 Right

**Table 3:** **Visuo-spatial and psychomotor testing**

	Simulation group (n = 4)	Control group (n = 6)	Mann–Whitney’s U-tests
Snowy pictures [mean(std dev)]	13.3 (5.6)	10 (4.8)	p = 0.285
Shape memory test	23.3 (4.6)	12.3 (4.6)	p = 0.010*
Card rotation test	21 (15.3)	6.67 (10.7)	p = 0.165
Pegboard - Sum Averages Right + Left + Both Hands	45.1 (8.0)	43.1 (5.3)	p = 0.522
Pegboard – Assembly	35.6 (7.8)	32.3 (5.7)	p = 0.240

Legend: Visuo-spatial testing using Snowy Picture, Shape Memory and Card Rotation Tests (Educational Testing Service) and psychomotor assessment using the Grooved Pegboard (Lafayette Instruments).

*p<0.05.

Video data corruption occurred during the recording of two participants’ ultrasound-guided axillary brachial plexus blockade, rendering assessment impossible (both in CG). As a results, a comparison of primary and secondary outcome measures involved 8 participants, with 4 in each group (Table 4). There was no statistically significant difference in clinical performance between the groups, as assessed using the sum of the GRS and CHECKLIST scores. There was also no difference in the secondary outcomes measured. No participant completed the performance of the block independently. Data relating to procedural times, number of needle passes and block success/failure were therefore not available. All candidates in both groups were adjudged by expert consensus to have "failed" in their performance of the block.

**Table 4:** **Primary and secondary outcome measures**

	Simulation group (n = 4)	Control group (n = 4)	Mann–Whitney’s U-tests
**GRS + CHECKLIST** [mean (std dev)]	32.9 (11.1)	31.5 (4.2)	p = 0.885
**GRS**	18.4 (5.8)	15.8 (1.7)	p = 0.561
**CHECKLIST**	14.5 (5.4)	15.8 (4.6)	p = 0.564

Participant assessment of content and delivery of the traditional training portion is shown in Table 5. Trainees in the SG rated elements of traditional training higher than CG participants. However, the magnitude of the differences tended to be low.

**Table 5:** **Participant assessment of content and delivery of the traditional training**

		Simulation group (n = 4)	Control group (n = 6)	Mann –Whitney’s U-tests
**Lecture**	*Quality of Speaker [median(range)]*	10 (10–10)	10 (8–10)	p = 0.224
	*Quality of Slides*	10 (10–10)	8 (8–9)	p = 0.005*
	*Potential to Learn*	10 (10–10)	8 (8–9)	p = 0.005*
**Cadaveric anatomy**	*Delivery of information*	10 (9–10)	8 (8–10)	p = 0.040*
	*Hands on Experience*	8 (7–10)	8 (6–10)	p = 0.904
**US scanning of volunteer**	*Delivery of information*	10 (10–10)	10 (9–10)	p = 0.221
	*Hands on Experience*	10 (9–10)	9 (5–10)	p = 0.069
**Tissue phantom**	*Delivery of Information*	10 (10–10)	10 (9–10)	p = 0.414
	*Hands on Experience*	10 (10–10)	9.5 (3–10)	p = 0.114

*p<0.05.

There was a trend towards a greater interval from commencement of training (traditional training session) to block performance in the Simulation group compared to that in the Control group; however this was not statistically significant [SG 24.5 (16.1) [mean (std dev)], CG 6.5 (6.0) respectively, p = 0.054].

The inter-rater reliability of the assessment of trainee performance by review of video was 89.3% (Range 83.7-93.9%) for checklist scores and 27.8% (Range 0–66.7%) for GRS scores. The Kappa for checklist scores was 0.749 (p < 0.01) indicating a good level of agreement [17], while the Kappa for GRS scores was not statistically significant (Kappa = 0.037, p = 0.628), indicating poor inter-rater reliability [17]. Table 4 compares i) sum of global rating scale plus checklist scores, ii) global rating scale scores, and iii) checklist scores between the two groups. Participant confidence did not differ statistically between the group 2 (2.45), and 2.83 (2.64) [mean (std dev)] in the Simulation and Control Groups respectively (p = 0.587).

## Discussion

We have described a methodology for assessing the effectiveness of simulator based training in improving novice trainee’s performance in a clinical setting, by means of a randomized control trial. Our pilot study found no difference in trainee performance between those who underwent standard training and those who received supplemental simulation-based training. This may have been due to a Type 2 error or to limitations of the prototype simulator (which led to early discontinuation of the trial). We recruited half our a priori defined group of 20 trainees, at one time point (the recruitment of trainee anesthetists in our region occurring biannually). Having trained this cohort, a planned natural point of interim analysis arose. Simulated sono-anatomy was subject to a number of limitations (e.g. clinically relevant muscles/tendons/fat were not modeled), resulting in relevant structures being presented against a relevantly homogenous background. There are two reasons for this; 1. The technical requirements to generate simulated structures, such as biceps or coracobrachialis muscles/tendons, would be significant and were beyond the resources of our team, and 2. The computational requirements to render these secondary structures accurately in real-time, as the user scanned the virtual arm, would be beyond the capacity of the available computer processing units. As a result, it is likely that the simulator allowed for identification of structures in an unrealistic fashion (i.e. lacked fidelity). Indeed, one participant in the SG commented that she would have preferred to attempt to perform the block at an interval closer to the traditional training session, where she had practiced scanning a real human volunteer. It is likely the simulator had a negative impact in teaching trainees sono-anatomy relevant to USgABPB. It is possible that this diminished any potential improvement in ultrasound-guided needle advancement. Following our interim analysis, we elected to suspend further recruitment as further use of our prototype simulator could have resulted in negative learning which could have negatively impacted on the care of real patients.

The recent Association for Medical Education in Europe (AMEE) Best Evidence in Medical Education (BEME) guide [18], highlighted outcome measures of education as one of the key areas requiring further research. This is the first study to look at the transfer of skills from VR simulation-based training to clinical practice, for an UGRA procedure. In their analysis of VR-based training for laparoscopic surgery, Sinitsky et al. [19] acknowledged that the science of setting proficiency levels is still ill-defined, describing it as "the most pressing issue". We chose to set proficiency levels based on a limited number of attempts by our group of experts (mean of first three attempts following initial familiarization). Sinitsky et al. [19] also recommended that laparoscopic procedural skills are best learnt through distributed not massed practice. A one day intensive hands-on course on UGRA is an example of massed practice, whereas distributed practice is spread over a greater period of time (shorter practice sessions with long intervals between sessions). In more general studies of the effectiveness of technology-enhanced learning on medical education, Cook [5, 20] also suggests distributed practice is more effective than massed practice. The same authors also found an association between individualized learning and better non-time based skills outcomes [20]. Following the initial familiarization session, trainee’s use of the simulator in this study was self-regulated. As a result, participants could train at a rate which best suited them and was distributed across a number of sessions over a number of days.

Our study is subject to a number of limitations. Firstly the prototype simulator used was inadequate, in terms of functionality, to meet the training requirements for teaching anesthesiologists, novice in UGRA. Our study sample was small and technical issues with video-recording decreased the size of the dataset acquired further. Inter-rater reliability between experts was poor for GRS scores. This is likely due to the relatively subjective nature of GRS assessment. This may have been improved by enhanced training on using the assessment tools. While inter-rater reliability was good for checklist scores, such tools are subject to a number of limitations. It is possible that an assessment tool which specifically captures clinically relevant errors would be more useful in assessing procedural skills. Such a tool would be particularly useful in providing formative feedback. In the absence of such a validated tool, we chose our primary outcome measure as a combination of GRS and checklist scores. The poor inter-rater reliability of the GRS component raises questions over the validity of our results. There was a difference in training time between the two groups. This difference related to the additional time it took participants in the simulation group to complete simulation training to the predefined proficiency level. It is possible that an improvement in performance in the SG could have been partially attributed to the increased training time, had this occurred. It is also possible that, in this novice population, elements of the traditional training were more important than those enhanced by the simulator training. In particular, when compared to the simulator-generated images, novices appeared overwhelmed by the amount of information they had to interpret in reality. The trend towards an increased interval from the traditional training to block performance in the SG may have had a negative impact on their performance. A number of elements of the traditional training session were rated lower by CG participants than by SG participants. This study does not look at cost of training as the simulator was under development and has no defined monetary value [21]. The simulator described utilizes haptic devices which are costly. Comparisons of haptic and non-haptic based in VR simulation has questioned the need for such devices when training laparoscopic surgical skills [22]. Future studies will need to address this question in training UGRA. Such analysis would have to include both the costs of the devices themselves and also the potential savings (e.g. those associated with faster training, more self-directed learning with lesser requirement for 1:1 teaching with trainers, lesser need for dedicated UGRA courses). The results of this study have informed the iterative development of the simulator. Ultrasound imagery in future prototypes will likely be based on real acquired ultrasound data [23] from which the simulator will be capable of rendering a real-time image. Rosenberg et al. have described mannequin-based simulators for ultrasound-guided regional anesthesia [24]. Based on our experience in this study, we have cautioned that the ultrasound imagery generated not be clinically relevant and could result in negative learning [25].

For the purpose of this study simulation-based training and traditional training were separated into two discrete components with no overlap (to control for possible confounding effects). In reality a more blended approach would be potentially more beneficial. This would likely lessen the negative impact of deficiencies of, for example, an inadequate training tool/simulator.

With increasing computational capacity and reduced cost, it is likely that simulation will move to a more personal environment where supervision is no longer a necessary component to the experience [26]. This may facilitate an individual gaining expertise through self-regulated deliberate practice. However establishing validity of such devices would be essential. The potential for a trainee to learn incorrect or dangerous techniques in an unsupervised simulated environment could have catastrophic results if transferred into the clinical domain [26]. To date, publications of simulation-based training in UGRA have largely been limited to descriptive pieces with few addressing transfer of skills into a clinical setting. In this study we attempted to address this deficit.

## Conclusions

We believe that the information acquired during this pilot study will be useful in performing future trials on learning efficacy associated with simulation-based training in procedural skills. In particular, confirmation of a degree of fidelity in the challenges rendered by a simulator is a pre-requisite to carrying out such a study. We believe that failure to do so, could result in spurious results due to factors other than the training or educational value of the simulation-based programme.

## Electronic supplementary material

Additional file 1: **Task Specific Checklist and Global Rating Scale for assessment Ultrasound Guided Axillary Brachial Plexus Block performance.** (DOCX 24 KB)

## Abbreviations

ABA: American board of anesthesiology
AMEE: Association for medical education in Europe
ASA: American society of anesthesiologists
BEME: Best evidence in medical education
CG: Control group
GRS: Global rating scale
LCD: Liquid crystal display
MOCA: Maintenance of certification in anesthesiology
SG: Simulator trained group
UGRA: Ultrasound-guided regional anesthesia
USgABPB: Ultrasound-guided axillary brachial plexus blockade
VR: Virtual reality.
