# Supplementary material for: The effect of simulation-based training on initial performance of ultrasound-guided axillary brachial plexus blockade in a clinical setting – a pilot study
Source: BMC Anesthesiol. 2014 Nov 26;14:110. doi: 10.1186/1471-2253-14-110 (PMC4384236; doi:10.1186/1471-2253-14-110)
Supplement: Supplementary file 1 — Additional file 1: Task Specific Checklist and Global Rating Scale for assessment Ultrasound Guided Axillary Brachial Plexus Block performance. (DOCX 24 KB) [file 12871_2014_359_MOESM1_ESM.docx]

Additional file 1

Appendix 1 - **Task Specific Checklist for Ultrasound Guided Axillary Brachial Plexus Block**

**CLEARLY IDENTIFIED OBSERVABLE BEHAVIOR**

**i.e. can be identified if seen by assessor on videotape**

Yes/No

**Positioning**

Exposure of the axilla ☐☐

The subjects dignity should be maintained

The arm should be out of the sleeve

Axilla and shoulder should be completely exposed

Positioning of arm ☐☐

Abduction - 90° at the shoulder

Flexion – flexion of arm at the elbow

External rotation – external rotation of arm

Patient comfort following positioning ☐☐

Positioning of Equipment

Ultrasound Screen ☐☐

Ultrasound machine screen should be in the same field of vision as the ultrasound probe

Sterile Trolley ☐☐

Sterile trolley should be within in arms distance and within the same field of vision as the ultrasound machine screen and the ultrasound probe

**Preparation**

Preparation of needle

22G gauge, 50mm Stimuplex needle (Standardized)

Needle flushed ☐☐

Preparation of Ultrasound Probe

Protection of probe ☐☐

Probe should be covered with either a sheath or a protective covering

Application of gel ☐☐

Gel can be applied to either axilla or ultrasound probe

**Block**

Preparation of Axilla

Antiseptic solution should be applied in the axilla ☐☐

Application of Ultrasound Probe

Orientation of probe ☐☐

Probe placed perpendicular to the arm in upper axilla ☐☐

Stabilizes transducer hand by resting gently on the patient ☐☐

Identification of Anatomical Structures

The participant will at this stage point at the ultrasound screen and identify the individual anatomical structures

Axillary Artery ☐☐

Axillary Vein/s ☐☐

The Axillary artery and vein should be identified via color flow analysis

Coracobrachialis muscle ☐☐

Musculocutaneous Nerve ☐☐

Median Nerve ☐☐

Ulnar Nerve ☐☐

Radial Nerve ☐☐

If using long axis approach maintain the needle in plane keeping whole needle in view at all times ☐☐

Deposition of Local Anesthetic

- For each nerve **(v) further dose injection** – the spread of Injectate should be visible on ultrasound screen

Nerve 1__________

- - 1. Needle tip is identified ☐☐
    2. Aspiration ☐☐
    3. Test Dose (spread of injectate identified) ☐☐
    4. Patient comfort on injection ☐☐
    5. Further dose injection^a^ ☐☐

Nerve 2__________

1. Needle tip is identified ☐☐
2. Aspiration ☐☐
3. Test Dose (spread of injectate identified) ☐☐
4. Patient comfort on injection ☐☐
5. Further dose injection^a^ ☐☐

Nerve 3 __________

1. Needle tip is identified ☐☐
2. Aspiration ☐☐
3. Test Dose (spread of injectate identified) ☐☐
4. Patient comfort on injection ☐☐
5. Further dose injection^a^ ☐☐

Nerve 4 __________

1. Needle tip is identified ☐☐
2. Aspiration ☐☐
3. Test dose (spread of injectate identified) ☐☐
4. Patient comfort on injection ☐☐
5. Further dose injection^a^ ☐☐

**Assessment**

Wound stabilization device removed ☐☐

Dressing/ cast should be removed before assessment

Patient should be asked about pain before removing device

Musculocutaneous Nerve

Sensory^b^ ☐☐

Lateral aspect of forearm should be checked for cold sensation

Motor ☐☐

Forearm Flexion

Radial Nerve

Sensory^b^ ☐☐

Posterior forearm, dorsum of hand, thumb, index and middle finger should be checked for cold sensation

Motor ☐☐

Wrist and finger Extension

Median Nerve

Sensory^b^ ☐☐

Anterior and medial aspect of forearm, thumb, index, middle and half of ring finger should be checked for cold sensation

Motor ☐☐

Flexion of lateral two fingers

Ulnar Nerve

Sensory^b^ ☐☐

Medial aspect of hand on the hypo-thenar eminence, little, ring and middle finger should be checked for cold sensation

Motor ☐☐

Thumb opposition or finger abduction

NOTES:

1. For each nerve (**v) further dose injection** – the spread of Injectate should be visible on ultrasound screen.
2. Regarding 13a-16a (sensory assessment) Assessment at one of listed sites is sufficientAppendix 2 - Generic Technical Skills Global Rating Scale

|  | | | | | | | |
| --- | --- | --- | --- | --- | --- | --- | --- |
| **Respect for Tissue** | **1** | **2** | | **3** | **4** | | **5** |
|  | Frequently used un-necessary force on tissue or caused damage | | Careful handling of tissue but occasionally caused inadvertent damage | | | Consistently handled tissue appropriately with minimal damage | |
| **Time and Motion** | **1** | **2** | | **3** | **4** | | **5** |
|  | Many un-necessary moves | | Efficient time/motion but some un-necessary moves | | | Clear economy of movement and maximum efficiency | |
| **Instrument Handling** | **1** | **2** | | **3** | **4** | | **5** |
|  | Repeatedly makes tentative or awkward moves with instruments by inappropriate use of instruments | | Competent use of instruments but occasionally appeared stiff or awkward | | | Fluid moves with instruments and no awkwardness | |
| **Knowledge of Instrument** | **1** | **2** | | **3** | **4** | | **5** |
|  | Frequently asked for wrong instruments or used inappropriate instrument | | Knew names of most instruments and used appropriate instruments | | | Obviously familiar with the instruments and their names | |
| **Flow of Procedure** | **1** | **2** | | **3** | **4** | | **5** |
|  | Frequently stopped procedure and seemed unsure of next move | | Demonstrated some forward planning with reasonable progression of procedure | | | Obviously planned course of procedure with effortless flow from one move to the next | |
| **Use of Assistants** | **1** | **2** | | **3** | **4** | | **5** |
|  | Consistently placed assistants poorly or failed to use | | Appropriate use of assistants most of the times | | | Strategically used assistants to the best advantage at all times | |
| **Knowledge of Procedure** | **1** | **2** | | **3** | **4** | | **5** |
|  | Deficient knowledge | | Knew all important steps of operation | | | Demonstrated familiarity with all aspects of operation/ procedure | |
| **Overall Performance** | **1** | **2** | | **3** | **4** | | **5** |
|  | Very poor | | Competent | | | Clearly superior | |

**Overall in this task, should the candidate**
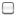
 **Pass**
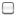
**Fail?**
